# Gel-immersion endoscopic detorsion for pediatric sigmoid volvulus

**DOI:** 10.1055/a-1858-4826

**Published:** 2022-06-24

**Authors:** Yasunori Yamamoto, Yoshiou Ikeda, Eiji Takeshita, Toshihiro Jogamoto, Takahiro Motoki, Mariko Eguchi, Yoichi Hiasa

**Affiliations:** 1 Endoscopy Center, Ehime University Hospital, Toon, Ehime, Japan; 2 Department of Gastroenterology and Metabology, Ehime University Graduate School of Medicine, Toon, Ehime, Japan; 3 Department of Pediatrics, Ehime University Graduate School of Medicine, Toon, Ehime, Japan

Pediatric sigmoid volvulus is a rare but emergency disease caused by abnormal twisting of the bowel along the mesenteric axis 1 2 . Water immersion colonoscopy has been reported to be effective for sigmoid volvulus because it minimizes colonic distension and improves visibility 3 4 . Gel-immersion endoscopy is a new method for securing the visual field during endoscopy 5 . The gel is better than water for removing contaminated bowel fluid and improving visibility. Moreover, the weight of the gel opens the twisting colon and facilitates volvulus passage. We present a case of pediatric sigmoid volvulus treated with gel-immersion endoscopic detorsion.

An 8-year-old girl with a double-outlet right ventricle was admitted to our hospital because of vomiting after an injection from a gastric fistula. She had no stool or flatus passage for two days, and her facial expression was anguished. Physical examination revealed a distended tympanic abdomen with high-pitched bowel sounds. Abdominal radiography revealed a huge dilated colonic loop ( Fig. 1 ). Computed tomography revealed bowel obstruction with swirling of the sigmoid colon, and sigmoid volvulus was suspected ( Fig. 2 ). A twisted intestine was confirmed at the sigmoid colon, but the poor endoscopic view caused by contaminated bowel fluid made endoscopic detorsion difficult. Therefore, we performed gel immersion endoscopy using ViscoClear (Otsuka Pharmaceutical Factory, Tokushima, Japan) ( Video 1 ). The injected gel provided a clear endoscopic view and helped assess intestinal ischemia ( Fig. 3 ). Moreover, in the left lateral decubitus position, the weight and pressure of the injected gel opened the twisted colon and facilitated volvulus passage ( Fig. 4 ). When the endoscope was passed through the torsion, a dilated intestinal lumen filled with gas and stool was observed, and endoscopic detorsion and decompression were successfully performed ( Fig. 5 ).

**Fig. 1:**
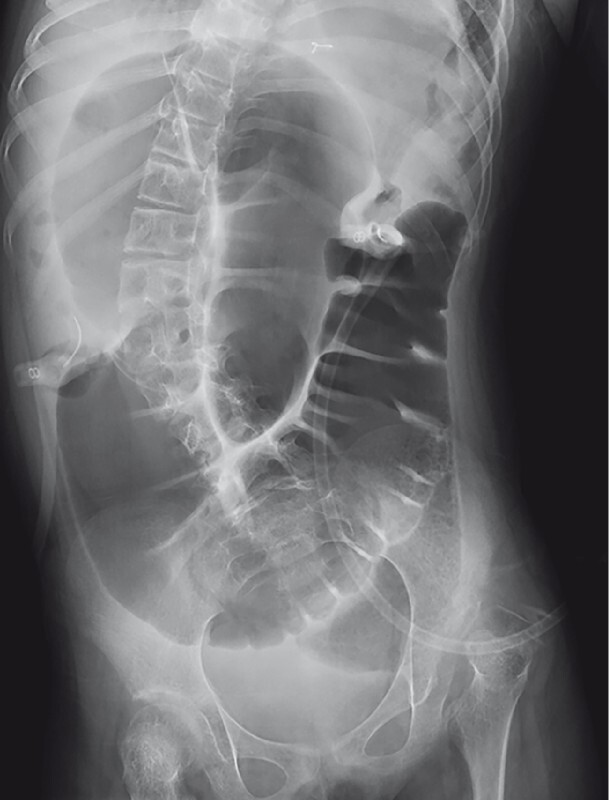
Abdominal radiograph shows a huge dilated colon.

**Fig. 2:**
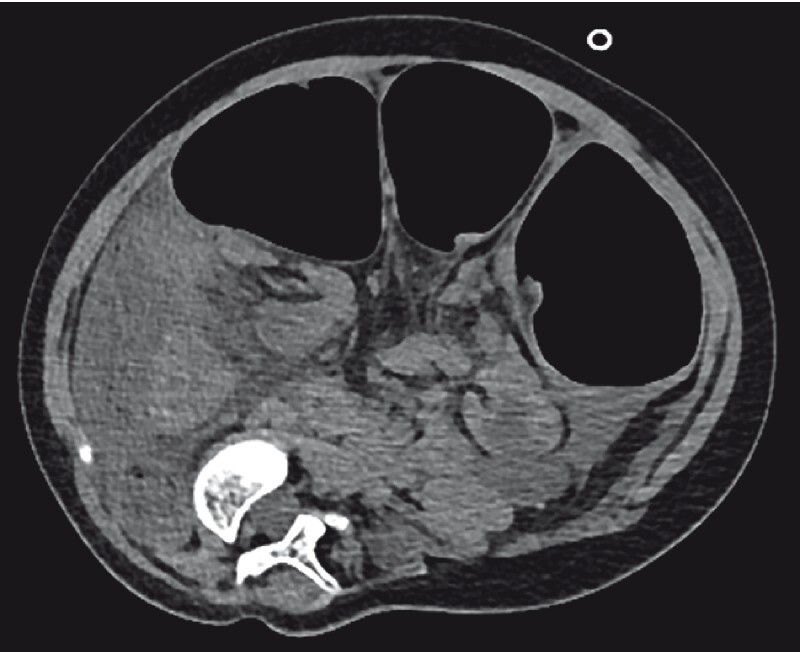
Abdominal computed tomography in the axial plane shows prestenotic dilatation of the sigmoid colon near the point of mesenterial rotation.

**Video 1** Successful endoscopic detorsion for pediatric sigmoid volvulus by using the gel-immersion method.


**Fig. 3:**
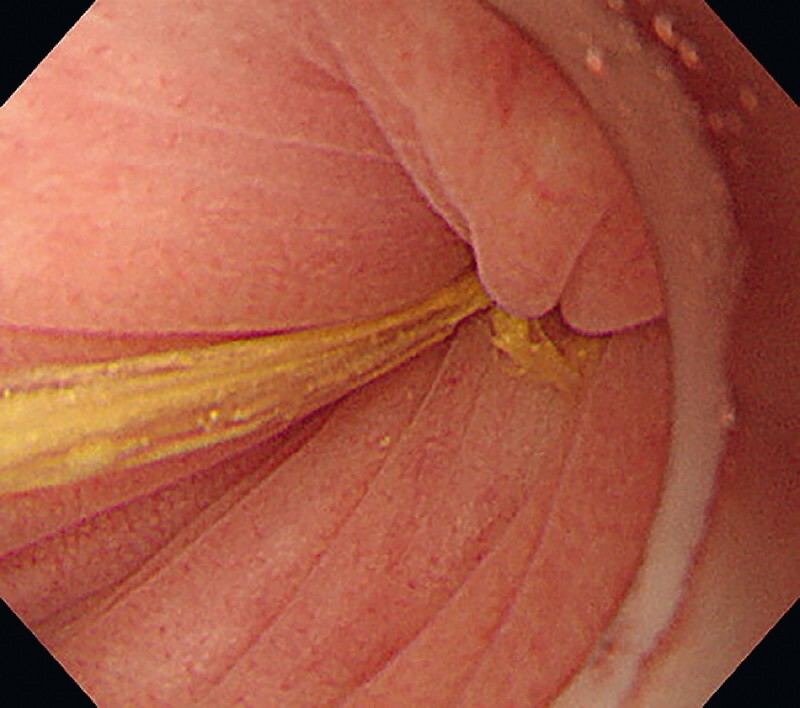
The twisted colon without ischemia was shown in a clear endoscopic view by the gel immersion method.

**Fig. 4:**
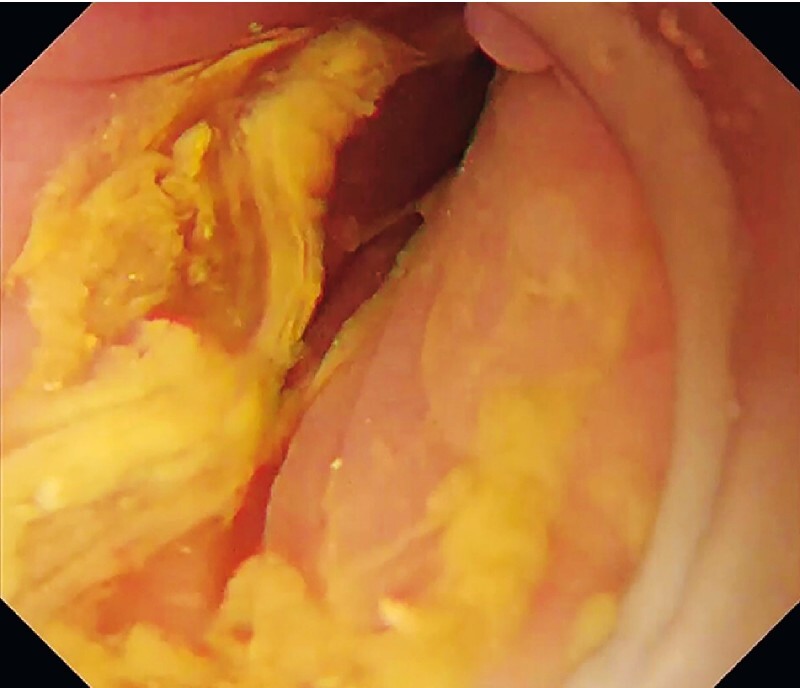
In the left lateral decubitus position, the pressure and weight of the injected gel opened the twisted colon.

**Fig. 5:**
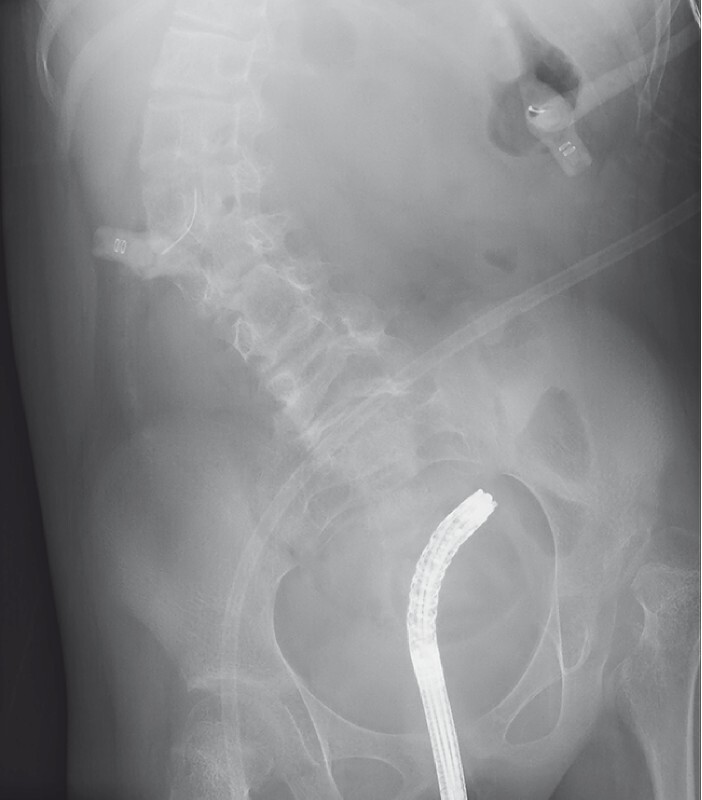
X-ray fluoroscopy showed that the dilated colon had disappeared.

This case study successfully employed gel-immersion endoscopy, which may be useful in the endoscopic detorsion of a sigmoid volvulus.

Endoscopy_UCTN_Code_TTT_1AQ_2AF

## Notes

Endoscopy E-Videos

https://eref.thieme.de/e-videos
*Endoscopy E-Videos* is an open access online section, reporting on interesting cases and new techniques in gastroenterological endoscopy. All papers include a high quality video and all contributions are freely accessible online. Processing charges apply (currently EUR 375), discounts and wavers acc. to HINARI are available. This section has its own submission website at https://mc.manuscriptcentral.com/e-videos
